# New evidence from China for the nature of the pterosaur evolutionary transition

**DOI:** 10.1038/srep42763

**Published:** 2017-02-16

**Authors:** Xiaoli Wang, Shunxing Jiang, Junqiang Zhang, Xin Cheng, Xuefeng Yu, Yameng Li, Guangjin Wei, Xiaolin Wang

**Affiliations:** 1 Institute of Geology and Paleontology, Linyi University, Linyi 276000, China; 2 Tianyu Natural History Museum of Shandong, Pingyi 273300, China; 3 Key Laboratory of Vertebrate Evolution and Human Origins, Institute of Vertebrate Paleontology and Paleoanthropology, Chinese Academy of Sciences, Beijing 100044, China; 4 Laboratory of Systematics and Taphonomy of Fossil Vertebrates, Department of Geology and Paleontology, Museu Nacional/UFRJ, Rio de Janeiro, RJ, 20940-040, Brazil; 5 Shandong Geological Sciences Institute, Jinan 250013, China; 6 University of Chinese Academy of Sciences, Beijing 100049, China

## Abstract

Pterosaurs are extinct flying reptiles, the first vertebrates to achieve powered flight. Our understanding of the evolutionary transition between basal, predominantly long-tailed forms to derived short-tailed pterodactyloids remained poor until the discovery of *Wukongopterus* and *Darwinopterus* in western Liaoning, China. In this paper we report on a new genus and species, *Douzhanopterus zhengi*, that has a reduced tail, 173% the length of the humerus, and a reduced fifth pedal digit, whose first phalange is ca. 20% the length of metatarsal III, both unique characters to Monofenestra. The morphological comparisons and phylogenetic analysis presented in this paper demonstrate that *Douzhanopterus* is the sister group to the ‘Painten pro-pterodactyloid’ and the Pterodactyloidea, reducing the evolutionary gap between long- and short-tailed pterosaurs.

Pterosaurs are extinct flying reptiles that achieved powered flight by developing an entirely distinctive anatomy comparable to any animals alive today. These reptiles are traditionally divided into two groups, short-tailed pterodactyloids and long-tailed ‘rhamphorhynchoids’^1^. In previous phylogenetic studies, the long-tailed ones have been shown to be a paraphyletic group^2^,^3^,^4^,^5^. However, our understanding of the evolutionary transition between the two groups was severly limited until the discovery of *Wukongopterus* and *Darwinopterus* from western Liaoning, China. In addition to *Kunpengopterus*, these transitional forms have derived cranial characters and primitive postcranial features^6^,^7^,^8^,^9^,^10^,^11^, alongside *Cuspicephalus scarfi*, from Dorset, England, which is also considered to be a basal monofenestratan^12^. Two further specimens, informally named ‘Rhamphodactylus’ and the ‘Painten pro-pterodactyloid’ have also been collected from the southern Franconian region of Germany and are relevant to this question. Both these pterosaurs exhibit mosaic characters, considered as the last step on the road from non-pterodactyloids to pterodactyloids^13^,^14^. Recently, *Allkaruen koi*, found in northern central Chubut of Argentina, was also reported to be the sister group of monofenestratan (Wukongopteridae + Pterodactyloidea) pterosaurs^15^. Here, we report on a new fossil specimen found in western Liaoning, China, which sheds new light on the origin of the Pterodactyloidea.

The new specimen reported here is housed in the Shandong Tianyu Museum of Nature; it was bought from a local farmer, who said that it had been collected in Toudaoyingzi, Jiangchang. However, based on the matrix surrounding the fossil as well as our own field work in this area, we are sure that this specimen was collected from Tiaojishan Formation in Linglongta. The age of these strata are disputed as some early researchers regarded them as ranging between the Middle and Late Jurassic^7^,^16^,^17^. Recently, however, this unit, especially the beds bearing *Darwinopterus*, have been considered early Late Jurassic in age^18^,^19^,^20^,^21^. Thus, the geological age of the specimen discussed here is considered to be early Late Jurassic^16^,^17^.

## Result

### Systematic palaeontology

Pterosauria Kaup, 1834, Monofenestrata Lü, Unwin, Jin, Liu et Ji, 2010, *Douzhanopterus zhengi* gen. et sp. nov.

#### Etymology

Douzhan, Chinese pinyin, the name of a buddha granted by Wukong, the Monkey King in the Chinese legend, indicating the relationship between this new pterosaur and other non-pterodactyloid monofenestratans, such as *Wukongopterus*, pterus, Greek, referring wings; species name in honor of Professor Xiaoting Zheng, who supported our research on this specimen.

#### Holotype

**Figure 1:**
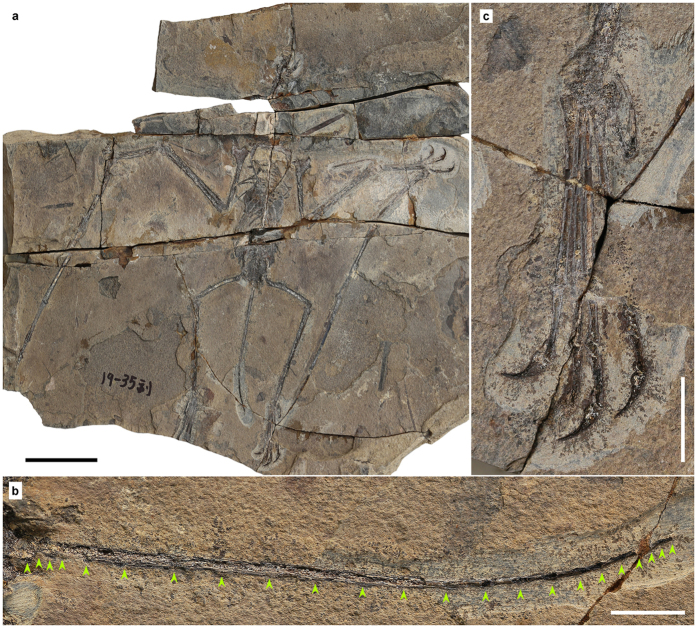
The holotype of *Douzhanopterus zhengi* gen. et sp. nov. (**a**) Part of the holotype; (**b**) close up of the tail, green arrows indicating the anterior and posterior ends of each caudal vertebra; (**c**) close up of the right foot. Scale bars are 50 mm, 10 mm, and 10 mm in (**a**,**b** and **c**), respectively.

**Figure 2:**
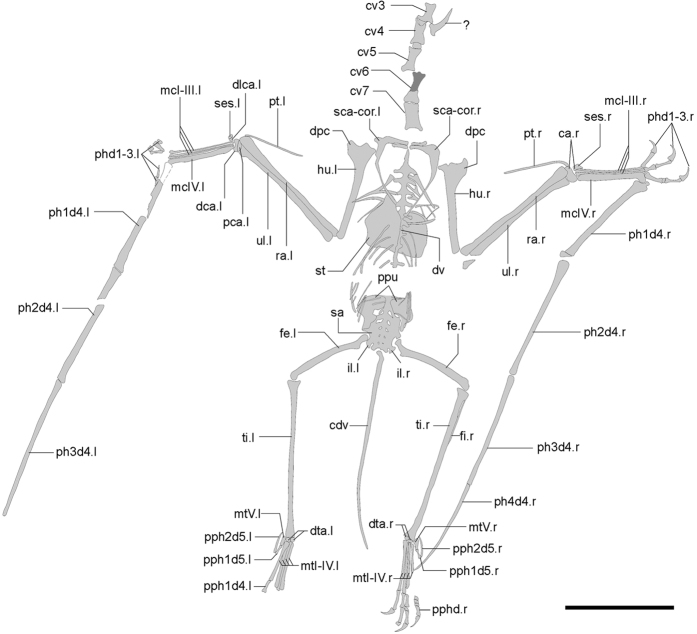
Line drawing of *Douzhanopterus zhengi* gen. et sp. nov. This illustration is based mostly on the part of the holotype, while dark grey areas are based on the counterpart. Abbreviations: ca, carpus; cdv, caudal vertebra; cv3–7, third to seventh cervical vertebra; dca, distal syncarpal; dlca, distal lateral carpal; dpc, deltopectoral crest; dta, distal tarsal; dv, dorsal vertebra; fe, femur; fi, fibula; hu, humerus; il, ilium; mcI-IV, metacarpal I-IV; mtI-V, metatarsal I-V; pca, proximal syncarpal; pphd, phalanges of pedal digit; pph1d4, first phalanx of pedal digit IV; pph1/2d5, first or second phalanx of pedal digit V; phd1–3, phalanges of manual digit I-III; ph1-4d4, first to fourth phalanx of manual digit IV; ppu, prepubis; pt, pteroid; ra, radius; sca-cor, scapulocoracoid; ses, sesamoid; st, sternum; sc, sacrum; ti, tibia; ul, ulna; l, left; r, right; ?, unknown element. Scale bar, 50 mm.

One complete postcranial skeleton housed at Shangdong Tianyu Museum of Nature, Pingyi, China (STM 19–35A & B) (Figs 1 and 2, Tables 1, 2 and 3).

#### Locality and Horizon

Linglongta, Jianchang, Liaoning, China; Daohugou Bed (or Tiaojishan Formation), Late Jurassic.

#### Diagnosis

Monofenestratan pterosaur diagnosed by autapomorphies including a reduced tail that is 173% the length of the humerus as well as a reduced fifth pedal digit, whose first phalange is ca. 20% length of metatarsal III. It can be further distinguished from other monofenestratan pterosaurs on the basis of the following combination of characters: the length of the mid-cervical 2.5–3.5 times of width; 22 caudal vertebrae with elongated zygapophyses and chevrons; pteroid over half length of ulna; tibia ca. 180% length of femur, and; fifth pedal digit V having two phalanges.

### Description

This new specimen is preserved in part and counterpart, and nearly all the postcranial elements are complete and articulated. Most of the bones are broken horizontally through the middle, and nearly all shafts of long bones are hollow inside, with thin cortices. Some cervical vertebrae are argillic, and only the outlines can be distinguished. The description below is mainly based on the part of the specimen (STM 19–35A, Figs 1 and 2). Elements only preserved on the counterpart (STM 19–35B) will be mentioned in the following text.

This new material comprises a small-sized pterosaur, with a wingspan of 0.74 m, twice the total length of coracoid, humerus, ulna, metacarpal IV, and four wing phalanges. Based on the fusion of syncarpals, scapulocoracoid, tibia and fibula, as well as the fact that the extensor tendon process is fused with the first wing phalange, this specimen is an osteologically mature individual^22^,^23^.

**Figure 3:**
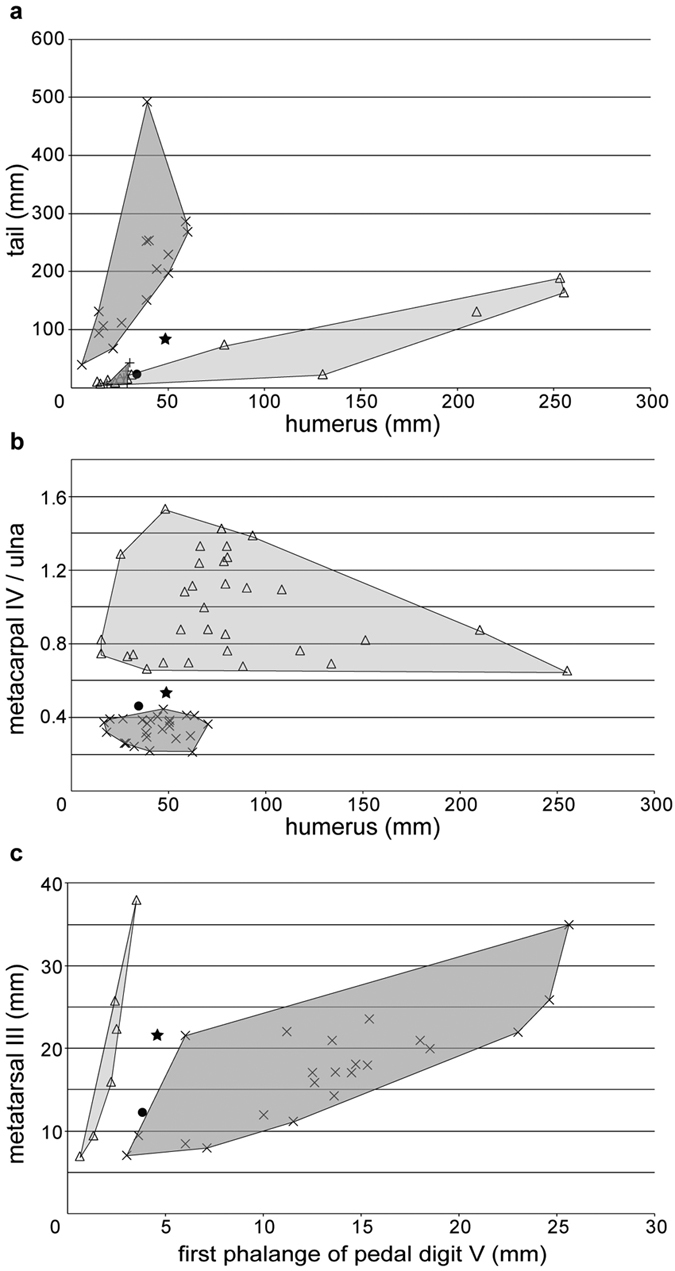
Comparison of *Douzhanopterus zhengi* gen. et. sp. nov. to other pterosaurs. (**a**) Relative length of the tail plotted against a proxy for body size (length of the humerus); (**b**) ratio of metacarpal to ulna against a proxy for body size (length of the humerus); (**c**) relative length of first phalange of pedal digit V plotted against a proxy for foot size (length of the metatarsal III). The data used here are listed in Supplementary Tables 1–3. Filled stars, *Douzhanopterus zhengi*; filled circles, ‘Painten pro-pterodactyloid’; triangles, pterodactyloids; positive crosses, anurognathids; oblique crosses, non-pterodactyloids (except anurognathids in (**a**)).

The first seven cervical vertebrae are preserved in STM 19–35, while additional elements preserved on the counterpart might include a fused atlas-axis. This section of the series has a distinct centrum condyle and posterodorsal directed neural spine, while the other part is obscured. Each mid-cervical vertebra has a pair of developed pre- and post-zygapophyses. The vertebrae are elongated, and the ratio of length to width is 2.5–3.5 (Table 1). There are nine dorsal vertebrae preserved, suggesting a total of 13 due to the distance between the last preserved dorsal vertebra and the sacrum. Six sacral vertebrae form a sacrum, and their transverse processes are directed lateroposteriorly, thicker than those of the of dorsal vertebrae. The caudal vertebrae of STM 19–35 are complete, even the distal most caudal, which was buried in the part before preparation. There are 22 caudal vertebrae, with a total length of 83.86 mm (Fig. 1b). These approximately elongate from the first to the sixth, and shorten to the last (Table 2). The zygapophyses and chevrons of the anterior caudal vertebrae are extremely elongated, at least three times length of centra (Fig. 1b), while the last elongated zygapophysis or chevron is present at the position of the sixteenth caudal. The last caudal vertebrae only have tiny centra. The length of tail is moderately reduced relative to the size of this animal (Fig. 3a; Supplementary Table 1), intermediate between long-tailed forms and short-tailed pterodactyloids.

The sternum of STM 19–35 is not complete, broken into two pieces. The cristospine is not preserved, but the left part of the sternal plate is nearly complete and no keel can be seen. The length of the sternum is similar to the width of the plate; the anterior margin is inclined posteriorly, and the posterior margin is nearly straight.

The scapula is slender. It is elongated and approximately 40% longer than coracoid (Table 3). No ventral expansion is present on the coracoid. The humerus is straight. It has a short deltopectoral crest, which is trapezoid and placed proximally, and it has a distinct ulnar crest, which directs posteriorly. No pneumatic foramen can be observed because of preservation, and the ulna is slightly thicker in mid-shaft than the radius. The pteroid is elongated, reaching half the length of ulna, and the outlines of the right carpals can be distinguished. The proximal and distal syncarpals formed and one small sesamoid attaches to the distal lateral carpal. The metacarpals are moderately elongated, around 65% the length of humerus and 53% the length of ulna, comprising a ratio between those basal and derived pterosaurs (Fig. 3b; Supplementary Table 2). Metacarpals I-III are articulated with the carpal as well as the wing metacarpal, and manual digits I-III bear large claws. The second wing phalange is the longest one; the first and third phalanges have similar length; the fourth one is slightly shorter than the others, but still over 80% length of the longest one (Table 3).

The pelvic girdles are fused with the sacrum; the preacetabular process is obscured but the postacetabular process is complete and short. Anterior to the acetabular fossa, two pieces of bones are present which we interpret as prepubes. These are longer than wide, and not fused in the middle. The femur of STM 19–35 is slightly curved and has a distinct head and constricted neck, forming a 150° angle with its shaft. The tibia is straight, and fused with the proximal and distal ends of the fibula, which reach up to 45% the length of the tibia (Table 3). Rectangular lateral and medial tarsals are preserved in each hind limb; metatarsal II is the longest, while metatarsal IV is shorter than metatarsals I-III. Metatarsal III is 31% the length of the tibia, while metatarsal V is the shortest (Table 3). The fifth pedal digit still has two phalanges; the first of these is straight while the second is slightly curved (Fig. 1c). The first pedal phalange is only 20% to 23% the length of metatarsal III, larger than those of pterodactyloids, but shorter than non-pterodactyloids (Fig. 3c; Supplementary Table 3).

### Systematics of *Douzhanopterus zhengi* sp. et gen. nov

**Figure 4:**
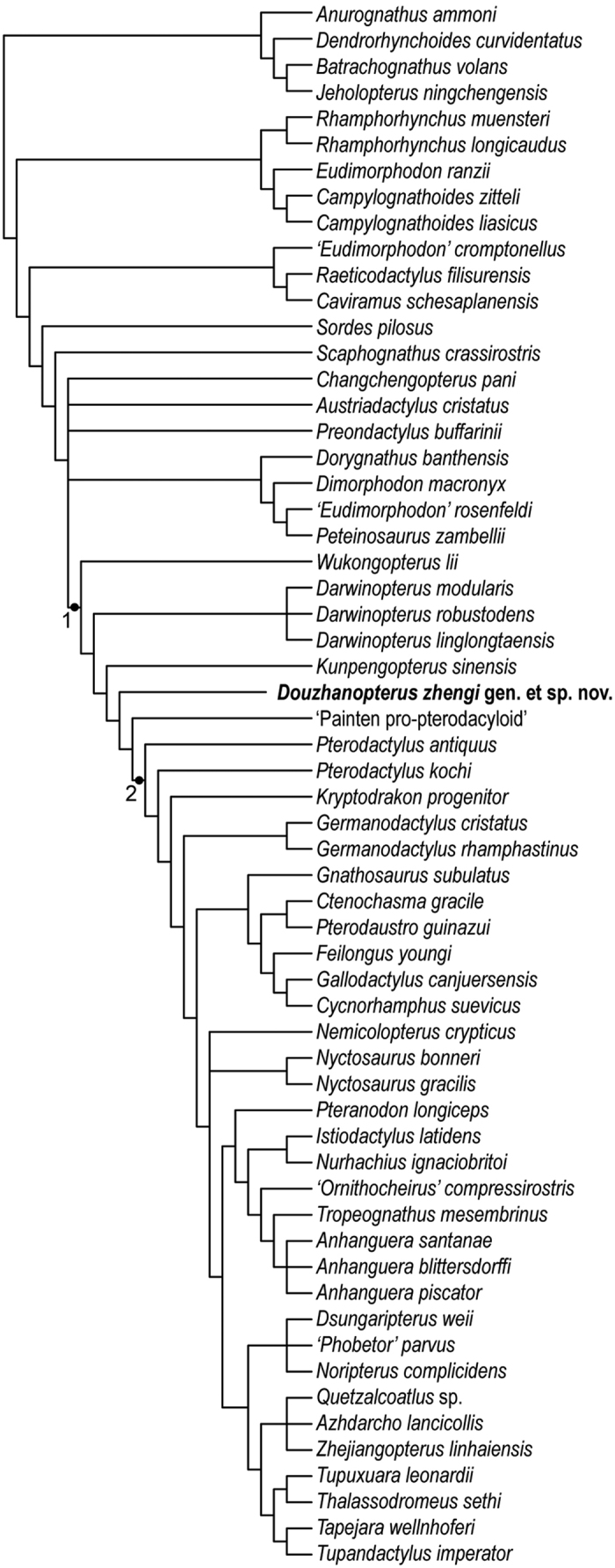
The reduced strict consensus tree of 114 MPTs each 253.393 steps in length. 1. Monofenestra; 2. Pterodactyloidea.

In order to access the position of *Douzhanopterus zhengi* gen. et sp. nov. within the Pterosauria, we carried out a new phylogenetic analysis (see Methods and Supplementary Methods). This analysis was mainly based on the previous work of Wang *et al*.^6^, consisting of 64 species (two outgroups and 62 ingroups) and 89 characters (including two continuous characters, modified from Andres *et al*.^5^). We also added *Darwinopterus, Kunpengopterus sinensis, Cuspicephalus scarfi*, the ‘Painten pro-pterodactyloid’, *Allkaruen koi, Changchengopterus pani, Douzhanopterus zhengi*, and *Kryptodrakon progenitor*, used TNT for the analysis, and generated 114 most parsimonious trees (MPTs), 253.383 steps in length (CI = 0.600; RI = 0.852). The strict consensus of these MPTs shows that the Monofenetrata and Pterodactyloidea are monophyletic, with the support of 12 and 18 characters, respectively (see the synapomorphies listed in the Supplementary Information). However, the relationship of non-pterodactyloid monofenestratans in this group are still not well-resolved. Through the comparison of 114 MPTs, two unstable taxa were identified, *Cuspicephalus scarfi* and *Allkaruen koi*. Both of them lie in basal positions of Monofenestrata. The reduced strict consensus tree without these two taxa is shown in Fig. 4. In this reduced tree, the ‘Painten pro-pterodactyloid’ is the sister group to the Pterodactyloidea, while *Douzhanopterus* occupies a more basal position, the sister group to the clade including the ‘Painten pro-pterodactyloid’ and the Pterodactyloidea. The ‘Wukongopteridae’ is not resolved as a monophyletic group in this analysis and *Wukongopterus lii* is the most basal monofenestran.

## Discussion

### Relationships among basal monofenestratans

Some basal monofenestratans have been considered transitional forms between non-pterodactyloids and pterodactyloids, including *Wukongopterus*^6^, *Darwinopterus*^7^,^8^,^9^,^10^, *Kunpengopterus*^8^, *Cuspicephalus*^12^, *Allkaruen*^15^, ‘Rhamphodactylus’^13^, and the ‘Painten pro-pterodactyloid’^14^. However, just the upper jaw of *Cuspicephalus* are preserved^12^, and it is absent in *Douzhanopterus*. The overlap with *Allkaruen* and *Douzhanopteus* is linited to cervical vertebrae^15^, which means that too limited information is available to distinguish these taxa from one another.

Compared with *Wukongopterus, Darwinopterus*, and *Kunpengopterus*^6^,^7^,^8^,^9^, *Douzhanopterus* occupies a derived position as it possesses a number of advanced postcranial ratios (Table 4). For example, the cervicals in *Douzhanopterus* are more elongated than those of the other three taxa, while the tail is less than half their length relative to body size. In addition, the fifth pedal digit of *Douzhanopterus* retains two much reducted pedal phalanges.

The description of ‘Rhamphodactylus’ is quite limited^13^, but the ‘Painten pro-pterodactyloid’ has been discussed in more detail^14^. Both these animals are young juvenile individuals because no fusion of postcranial elements can be found. They are in the same size and have quite similar ratios of postcranial elements, although the measurements of ‘Rhamphodactylus’ were calculated in this study based on figures. Indeed, these two were not treated as the same taxon because of differences in their geological ages^14^. Compared with the new Chinese material, both these enigmatic taxa share the presence of an elongated metacarpal IV (62–66% the length of the humerus, and 46–53% the length of the ulna), reduced first (20% to 31% the length of metatarsal III) and second phalanges of pedal digit V, as well as a reduced tail (0.8–1.7 the length of the humerus) with elongated zygapophyses and chevrons, demonstrating the transitional position of these three specimens between other non-pterodactyloid mononfenestratans and pterodactyloids. However, *Douzhanopterus* varies compared to the two German specimens in a number of postcranial ratios (Table 4). For example, the new Chinese taxon has a larger pteroid/ulna length ratio than that of the two German specimens (Table 4), while its tibia/femur length ratio is much larger than that of the other two non-pterodactyloid monofenestratans (Table 4). *Douzhanopterus* and the ‘Painten pro-pterodactyloid’ have two reduced phalanges of the fifth pedal digit relative to other non-pterodactyloids, while our new Chinese taxon boasts a smaller ratio between the first phalange of pedal digit V to metatarsal III (20% and 23%) compared to the ‘Painten pro-pterodactyloid’ (31%). The tails of the two German pterosaurs are also incomplete, lacking their distal-most ends. Thus, assuming that these distal-most caudal vertebrae exhibit the same average lengths as the anterior parts of the ‘Painten pro-pterodactyloid’, the whole tail of the ‘Painten pro-pterodactyloid’ would have a maximum length of 29.6 mm, shorter than its humerus. *Douzhanopterus* also has 22 caudal vertebrae, more than the ‘Painten pro-pterodactyloid’ which has an estimated 17^14^; the former also has much larger tail/humerus length ratio, nearly twice than that of the latter, but still less than half that of other non-pterodactyloid monofenestratans (Table 4). The centra, rod-like zygaporphyses, and chevrons of the caudal vertebrae in *Douzhanopterus* are slightly more elongated than those of the German specimens, but not so much as in other non-pteroadctyloids. Based on our as yet unpublished study of young basal monofenestratan specimens, tail length relative to body size changes, but the number of caudal vertebrae and the elongation of fifth pedal digits remain stable during ontogeny. Hence, *Douzhanopterus* has a primitive tail but a slightly derived foot relative to the German specimens; thus, as the result of our phylogenetic analysis, *Douzhanopterus* lies in a more basal position than the ‘Painten pro-pterodactyloid’.

The discovery of *Douzhanopterus* enhances our understanding of the origin of the Pterodactyloidea. Indeed, *Wukongopterus, Darwinopterus*, and *Kunpengopterus*, known on the basis of abundant specimens from the early Oxfordian (160–161 Ma)^17^, are the first step in this transition, and evidence mainly cranial changes^6^,^7^,^8^,^9^. Specimens of *Douzhanopterus*, however, from the same deposits, are much rarer than these taxa, which may indicate that these basal monofenestratans were dominant at the time while *Douzhanopterus* occupied an inferior position. Hence, monofenestratans including *Wukongopterus* and *Darwingopterus*, may first appear further back in time into the Middle or even Early Jurassic^15^, becoming more diverse and successful during the early Late Jurassic. We hypothesize that *Douzhanopterus* likely originated adjacent to, or around this time.

Basing on a study of *Darwinopterus modularis*, modular evolution was first proposed by Lü *et al*.^7^. Building on this, the intermediate characters seen in *Douzhanopterus* can also be divided into two modules, an anterior part encompassing the postcranium, mainly cervical vertebrae and metacarpals, and a posterior part, mainly encompassing caudal vertebra and feet. In the anterior part, elongation of cervicals is seen, as was the case in *Wukongopterus* and *Darwinopterus*, whose cervical length/diameter ratios are no more than 2.5^6^,^7^,^8^,^9^. In *Douzhanopterus*, this ratio becomes slightly larger, suggesting that this trend in cervical elongation was maintained. This effect was also seen in the metacarpals^6^,^8^, and elongation was again maintained in *Douzhanopterus*. The posterior parts of the skeleton change significantly; tail length relative to body size in *Douzhanopterus* was reduced by at least 50% compared to *Wukongopterus* and *Darwinopterus*. The new Chinese pterosaur also possesses by far the oldest record of a reduced fifth digit, and it is shorter than third of that in *Wukongopterus* and *Darwinopterus* relative to its body size. This is corroborated by the fact that both ‘Rhamphodactylus’ and the ‘Painten pro-pterodactyloid’ were collected from early Late Tithonian-aged deposits (ca. 150 Ma)^14^, about 10 million years younger than the age of *Douzhanopterus*. These two German specimens have shorter tails than *Douzhanopterus*, which are quite similar to pterodactyloids. The other postcranial changes between *Douzhanopterus* and the two German pterosaurs are not significant, and even some characters, such as reduction of fifth pedal digits and elongation of metacarpals, are more primitive in the latter specimens than in *Douzhanopterus*. Some researchers have suggested that modularity may play an important role at one anatomical level, while at a finer level, evolution within a module may follow a mosaic pattern^15^. The distinctions we report between *Douzhanopterus* and two the German pterosaurs are consistent with this hypothesis.

## Methods

The phylogenetic analysis was conducted with the software Tree Analysis Using New Technology (TNT) version 1.1^24^. The ‘Max.trees’ setting was set to 10,000 and zero-length branches were collapsed using the rule ‘min. length = 0’. Two ‘Traditional search’ rounds were used, first starting with Wagner trees and duplicating 1,000 times, while the second round was built on trees from memory. The command ‘best’ was used to filter the trees for the best score, and all others were set as default. As discussed in the text, we added *Douzhanopterus zhengi* gen. et sp. nov., the ‘Painten pro-pterodactyloid’, *Allkaruen koi, Kryptodrakon progenitor, Cuspicephalus scarfi, Changchengopterus pani, Darwinopterus modularis, D. linglongtaensis, D. robustdens*, and *Kunpengopterus sinensis* to the matrix of Wang *et al*.^6^, as well as two continuous characters, the ratio between the metacarpal/humerus length and caudal length/diameter (modified from Andres *et al*.^5^). Another new character relating to the length of the first phalanx of pedal digit V compared to metatarsal III was added as character 88, while characters that relate to the humerus/metacarpal-IV, femur/metacarpal-IV, and ulna/metacarpal-IV length ratios as used by Wang *et al*.^6^were deleted, to avoid artificially upweighting the ratio of metacarpal IV to other elements. We also re-coded *Wukongopterus lii* for character 50; this character relates to peg-like teeth. In the holotype and the only specimen of *Wukongopterus lii*, the skull is incomplete, and there are less than 15 peg-like teeth preserved on either side. Thus, the inferred number of teeth for each side in upper and lower jaws are 16 and 12, respectively^6^. If this is correct, 16 teeth should be scored as state 2 and 12 teeth is state 1; we consider that it is better to choose the two states, but not just state 2. The complete characters and synapomorphies of nodes present in the strict consensus tree are listed in Supplementary Methods. The file with complete matrix, which can be run directly in TNT, is provided as Supplementary Dataset.

## Additional Information

**How to cite this article**: Wang, X. *et al*. New evidence from China for the nature of the pterosaur evolutionary transition. *Sci. Rep.* **7**, 42763; doi: 10.1038/srep42763 (2017).

**Publisher's note:** Springer Nature remains neutral with regard to jurisdictional claims in published maps and institutional affiliations.

## Supplementary Material

Supplementary Dataset 1

Supplementary Information

## Figures and Tables

**Table 1:** Measurements of cervical vertebrae in the holotype of *Douzhanopterus zhengi* gen. et sp. nov. (in mm).

	Length (prz-poz)	Width in the middle
Cervical 3	8.42*	—
Cervical 4	10.12	4.03
Cervical 5	12.85	3.62
Cervical 6	14.25†	—
Cervical 7	13.07	3.83

^*^Preserved length.

^†^Estimated length.

**Table 2:** Measurements of caudal vertebrae in the holotype of *Douzhanopterus zhengi* gen. et sp. nov. (in mm).

	Length of centrum	Diameter		Length of centrum	Diameter
1	1.36	1.50	12	6.4	0.82
2	1.24	1.53	13	4.78	0.9
3	3.14	1.26	14	5.04	0.69
4	3.46	1.26	15	4.31	0.63
5	4.98	1.33	16	3.96	0.6
6	7.76	1.2	17	2.87	0.67
7	6.26	1.07	18	2.74	0.71
8	7.51	1.19	19	2.16	0.58
9	6.67	1.25	20	1.88	—
10	5.76	1.15	21	1.62	0.54
11	6.35	0.93	22	1.5	0.53

**Table 3:** Measurements of postcranial elements in the holotype of *Douzhanopterus zhengi* gen. et sp. nov. (in mm).

	Left	Right
scapular	32.64	33.15
coracoid	23.91	21.80
humerus	48.49	46.95*
radius/ulna	59.49	59.25*
metacarpal IV	31.72	31.86
pteroid	30.53	30.88
first phalange of manual digit IV	53.26	49.32*
second phalange of manual digit IV	55.26*	56.15*
third phalange of manual digit IV	52.70*	53.19
fourth phalange of manual digit IV	—	45.54*
femur	41.21	39.27
tibia	69.85	70.52
fibula	—	31.44
metatarsal III	21.58	21.86
metatarsal IV	17.61	17.34*
metatarsal V	4.94	5.08
first phalange of pedal digit V	4.24	4.54
second phalange of pedal digit V	9.27	9.79

^*^Preserved length.

**Table 4:** Comparison of postcranial ratios among basal monofenestrans.

	mcIV/hu	mcIV/ul	pt/ul	fe/hu	ti/fe	pph1d5/mtIII	ta/hu	Sources
*Douzhanopterus zhengi* gen. et sp. nov holotype	0.65	0.53	0.51	0.81	1.80	0.20; 0.23	1.73	Measured by authors
‘Rhamphodactylus’	0.65*	0.50*	0.34*	0.95*	1.24*	—	—	13
‘Painten pro-pterodactyloid’	0.63	0.46	0.34	0.95	1.18	0.31	<0.87†	14
*Wukongopterus lii* holotype	0.59	0.37	—	0.86	1.59	0.75	>3.92	6, pph1d5 and tail measured by authors
*Kunpengopterus sinensis* holotype	0.64†	0.39†	0.44†	1.14†	1.32†	0.50	—	8, pph1d5 measured by authors
*Darwinopterus modularis* referred specimen	0.59	0.41	0.41	0.91	1.35	0.85	4.66*	7
*Darwinopterus linglongtaensis* holotype	0.59	0.39	0.47	1.00	1.24	0.72	—	8, pph1d5 measured by authors
*Darwinopterus robustodens* holotype	0.60	0.38	—	0.86	1.40	0.86	>4.60	9
*Pterodactylus antiquus* holotype	1.11	0.74	—	1.10	1.39	0.14	0.67	25
*Sinopterus dongi* holotype	1.64	1.09	0.43	1.28	1.41	—	—	26

*Pterodactylus antiquus* and *Sinopterus dongi* represent the Pterodactyloidea, giving an indication of polarity.

Abbreviation: fe, femur; hu, humerus; mcIV, metacarpal IV; mtIII, metatarsal III; pph1d5, first phalnage of pedal digit V; pt, pteroid; ti, tibia; ta, tail; ul, ulna.

^*^Calculated on length from figures.

^†^Calculated on estimated length.
