# Supplementary material for: New evidence from China for the nature of the pterosaur evolutionary transition
Source: Sci Rep. 2017 Feb 16;7:42763. doi: 10.1038/srep42763 (PMC5311862; doi:10.1038/srep42763)
Supplement: Supplementary Information [file srep42763-s2.doc]

**New evidence from China for the nature of the pterosaur evolutionary transition**

Xiaoli Wang^1,2^, Shunxing Jiang*^3^, Junqiang Zhang^1,2^, Xin Cheng^3,4^, Xuefeng Yu^5^, Yameng Li^1,2^, Guangjin Wei^1,2^, Xiaolin Wang^3,6^

1 Institute of Geology and Paleontology, Linyi University, Linyi 276000, China.

2 Tianyu Natural History Museum of Shandong, Pingyi 273300, China.

3 Key Laboratory of Vertebrate Evolution and Human Origins, Institute of Vertebrate Paleontology and Paleoanthropology, Chinese Academy of Sciences, Beijing 100044, China.

4 Laboratory of Systematics and Taphonomy of Fossil Vertebrates, Department of Geology and Paleontology, Museu Nacional/UFRJ, Rio de Janeiro, RJ, 20940-040, Brazil.

5 Shandong Geological Sciences Institute, Jinan 250013, China.

6 University of Chinese Academy of Sciences, Beijing 100049, China.

Correspondence and requests for materials should be addressed to S.J. (email: jiangshunxing@ivpp.ac.cn).

1. Supplementary methods.

2. Supplementary tables 1-3

3. Supplementary references

Supplementary methods

CHARACTER LIST

Continuous characters

0. Caudal vertebra, length relative to diameter: continuous, values from 0.857 to 9.932 scaled from 0 to 1 (modified from Andres et al. 2014^5^)

1. Metacarpal IV, length relative to humerus length: continuous, values from 0.131 to 2.996 scaled from 0 to 1 (modified from Andres et al. 2014^5^)

Numeric characters

2. Dorsal margin of the skull:

0 - straight or curved downward

1 - concave

2 - only rostrum curved upward

3. Upper and lower jaw:

0 - laterally compressed

1 - comparatively broad

4. Rostral part of the skull anterior to the external nares:

0 - reduced

1 - elongated (less than half of skull length)

2 - extremely elongated (more than half of skull length)

5. Rostral end of premaxillae/maxillae downturned:

0 - absent

1 - present

6. Position of the external naris:

0 - above the premaxillary tooth row

1 - displaced posterior to the premaxillary tooth row

7. Process separating the external nares:

0 - broad

1 - narrow

8. Dorsoventrally compressed and elongated naris

0 - absent

1 - present

9. Naris size relative antorbital fenestra

0 - naris smaller than antorbital fenestra

1 - naris larger than antorbital fenestra

2 - both very reduced (slit-like)

10. Naris and antorbital fenestra:

0 - separated

1 - confluent, shorter than 45% of the skull length

2 - confluent, longer than 45% of the skull length

11. Anorbital fenestra, shape

0 - eliptical or ovoid

1 - triangular, with base and height subequal

2 - triangular with height larger than base

3 - very elongated anteroposteriorly

12. Orbit comparatively small and positioned very high in the skull:

0 - absent

1 - present

13. Orbit pear-shaped:

0 - absent

1 - present

14. Position of the orbit relative to the nasoantorbital fenestra (naris + antorbital fenestra):

0 - same level or higher

1 - orbit lower than the dorsal rim of the nasoantorbital fenestra

15. Suborbital opening:

0 - absent

1 - present

16. Premaxillary sagittal crest, position:

0 - absent

1 - confined to the anterior portion of the skull

2 - starting anterior to the anterior margin of the nasoantorbital fenestra, not reaching the skull roof above the orbit

3 - starting anterior to the anterior margin of the nasoantorbital fenestra, extending beyond occipital region

4 - starting at about the anterior margin of the nasoantorbital fenestra, reaching the skull roof above the orbit but not extending over the occipital region

5 - starting close or at the anterior portion of the skull and extended over the occipital region

6 - starting close or at the anterior portion of the skull, reaching orbit but not extended over the occipital region

7 - starting at the posterior half of the nasoantorbital fenestra.

17. Premaxillary sagittal crest shape:

0 - striated, low with a nearly straight dorsal margin

1 - striated, high with a nearly straight dorsal margin

2 - striated, high, spike-like

3 - round dorsal margin, bladeshaped

4 - smooth, moderately expanded anteriorly and forming a low rod-like extension posteriorly

5 - smooth, very expanded anteriorly and forming a low rod-like extension posteriorly

6 - smooth, starting low anteriorly and very expanded posteriorly

18. Expansion of the premaxillary tip:

0 - absent

1 - present, with premaxillary end high

2 - present, with premaxillary end dorsoventrally flattened.

19. Posterior ventral expansion of the maxilla:

0 - absent

1 - present

20. Maxilla-nasal contact

0 - broad

1 - narrow

2 - absent

21. Nasal process:

0 - absent

1 - placed laterally, long, straight, and directed ventrally (not fused with maxillae)

2 - placed laterally, reduced

3 - placed medially, long

4 - placed medially, reduced

5 - placed laterally, short and directed anteriorly

22. Foramen on nasal process:

0 - absent

1 - present

23. Anterior process of jugal rodlike and deflected dorsally

0 - absent

1 - present

24. Lacrimal process of the jugal:

0 - broad

1 - thin, subvertical

2 - thin, strongly inclined posteriorly

25. Bony frontal crest:

0 - absent

1 - low and blunt

2 - low and elongated

3 - high and expanded posteriorly

26. Bony parietal crest:

0 - absent

1 - present, blunt

2 - present, laterally compressed and posteriorly expanded, with a rounded posterior margin.

3 - present, constituting the base of the posterior portion of the cranial crest.

27. Posterior region of the skull rounded with the squamosal displaced ventrally:

0 - absent

1 - present

28. Position of the quadrate relative to the ventral margin of the skull:

0 - vertical or subvertical

1 - inclined about 120° backwards

2 - inclined about 150° backwards

29. Position of the articulation between skull and mandible:

0 - under the posterior half of the orbit or further backwards

1 - under the middle part of the orbit

2 - under the anterior half of the orbit

30. Helical jaw joint:

0 - absent

1 - present

31. Supraoccipital:

0 - does not extend backwards

1 - extends backwards

32. Foramen pneumaticum piercing the supraoccipital:

0 - absent

1 - present

33. Expanded distal ends of the paroccipital processes:

0 - absent

1 - present

34. Basisphenoid:

0 - short

1 - elongated

35. Palatal ridge:

0 - absent

1 - discrete, tapering anteriorly

2 - strong, tapering anteriorly

3 - strong, confined to the posterior portion of the palate

36. Maxilla excluded from the internal naris:

0 - absent

1 - present

37. Opening between pterygoids and basisphenoid (interpterygoid opening):

0 - absent or very reduced

1 - present and larger than subtemporal fenestra

2 - present but smaller than subtemporal fenestra

38. Large distinct foramina (cup-shaped structures) on the lateral side anterior portion of the dentary

0 - absent

1 - present

39. Mandibular symphysis:

0 - absent or very short

1 - present, at least 30% of mandible length

40. Anterior tip of the dentary downturned:

0 - absent

1 - present

41. Tip of the dentary projected anteriorly:

0 - absent

1 - present

42. Dentary bony sagittal crest:

0 - absent

1 - blade-like and short, placed anteriorly

2 - massive and deep.

43. Distinctively posteriorly oriented articular and retroarticular process

0 - absent

1 - present

44. Position and presence of teeth:

0 - teeth present, evenly distributed along the jaws

1 - teeth absent from the anterior portion of the jaws

2 - teeth confined to the anterior part of the jaws

3 - jaws toothless

45. Largest maxillary teeth positioned posteriorly:

0 - absent

1 - present

46. Variation in the size of the anterior teeth with the 5th and 6th smaller than the 4th and 7th:

0 - absent

1 - present

47. Teeth with a broad and oval base:

0 - absent

1 - present

48. Multicusped teeth:

0 - absent

1 - present

49. Teeth finely serrated

0 - present

1 - absent

50. Peg-like teeth:

0 - absent

1 - present, 15 less on each side of the jaws

2 - present, more than 15 on each side of the jaws

51. Long slender teeth:

0 - absent or less than 150

1 - present, more than 150

52. Laterally compressed and triangular teeth

0 - absent

1 - present

53. Notarium:

0 - absent

1 - present

54. Atlas and axis:

0 - unfused

1 - fused

55. Postexapophyses on cervical vertebrae:

0 - absent

1 - present

56. Lateral pneumatic foramen on the centrum of the cervical vertebrae:

0 - absent

1 - present

57. Midcervical vertebrae:

0 - short, sub-equal in length

1 - elongated

2 - extremely elongated

58. Cervical ribs on midcervical vertebrae:

0 - present

1 - absent

59. Neural spines of the midcervical vertebrae:

0 - tall, blade-like

1 - tall, spike-like

2 - low, blade-like

3 - extremely reduced or absent

60. Number of caudal vertebrae:

0 - more than 15

1 - 15 or less

61. Caudal vertebrae with elongated zygapophyses forming rod-like bony processes

0 - absent

1 - present

62. Length of the scapula:

0 - subequal or longer than coracoid

1 - scapula shorter than coracoid (1 > sca/cor > 0.80)

2 - substantially shorter than coracoid (sca/cor ≤ 0.80)

63. Proximal surface of scapula:

0 - elongated

1 - sub-oval

64. Shape of scapula

0 - elongated

1 - stout, with constructed shaft

65. Coracoidal contact surface with sternum:

0 - no developed articulation surface

1 - articulation surface flattened, lacking posterior expansion

2 - articulation surface oval, with posterior expansion

66. Deep coracoidal flange:

0 - absent

1 - present

67. Broad tubercle on ventroposterior margin of coracoid:

0 - absent

1 - present

68 - Cristospine:

0 - absent

1 - shallow and elongated

2 - deep and short

69. Proportional length of the humerus relative to the femur (hu/fe):

0 - hu/fe ≤0.80

1 - 1.4 ≥ hu/fe ≥ 0.80

2 - hu/fe > 1.40

70. Proportional length of the humerus plus ulna relative to the femur plus tibia (hu+ul/fe+ti):

0 - humerus plus ulna about 0.80% or less of femur plus tibia length

(hu+ul/fe+ti < 0.80)

1 - humerus plus ulna larger than 0.80% of femur plus tibia length

(hu+ul/fe+ti > 0.80)

71. Pneumatic foramen on the ventral side of the proximal part of the humerus

0 - absent

1 - present

72. Pneumatic foramen present on dorsal side of the proximal part of the humerus

0 - absent

1 - present

73. Deltopectoral crest of the humerus:

0 - reduced, positioned close to the humerus shaft

1 - enlarged, proximally placed, with almost straight proximal margin

2 - subrectangular, extending down the humerus shaft for at least 30% of humerus length

3 - distally expanded

4 - enlarged, hatchet shaped, proximally placed

5 - enlarged, hatched shaped, positioned further down the humerus shaft

6 - enlarged, warped

7 - long, proximally placed, curving ventrally

74. Medial (= ulnar) crest of the humerus:

0 - absent or reduced

1 - present, directed posteriorly

2 - present, massive, with a developed proximal ridge

75. Distal end of the humerus:

0 - oval or D-shaped

1 - subtriangular

76. Diameter of radius and ulna:

0 - subequal

1 - diameter of the radius about half that of the ulna

2 - diameter of the radius less than half that of the ulna

77. Distal syncarpals:

0 - unfused

1 - fused in a rectangular unit

2 - fused in a triangular unit

78. Pteroid:

0 - absent

1 - shorter than half the length of the ulna

2 - longer that half the length of the ulna

79. Metacarpals I - III:

0 - articulating with carpus

1 - metacarpal III articulates with carpus, metacarpals I and II reduced

2 - not articulating with carpus

80. Proportional length of the first phalanx of manual digit IV relative to the metacarpal IV (ph1d4/mcIV):

0 - both small and reduced

1 - both enlarged with ph1d4 over four times the length of mcIV (ph1d4/mcIV>4.0)

2 - both enlarged with ph1d4 between two and four times the length of mcIV (4.0 ≤ ph1d4/mcIV ≤ 2.0)

3 - both enlarged with ph1d4 less than two times the length of mcIV (ph1d4/mcIV<2.0)

81. Proportional length of the first phalanx of manual digit IV relative to the tibiotarsus (ph1d4/ti):

0 - ph1d4 reduced

1 - ph1d4 elongated and less than twice the length of ti (ph1d4/ti smaller than 2.00)

2 - ph1d4 elongated about or longer than twice the length of ti (ph1d4/ti subequal/larger than 2.00)

82. Proportional length of the second phalanx of manual digit IV relative to the first phalanx of manual digit IV (ph2d4/ph1d4):

0 - both short or absent

1 - elongated with second phalanx about the same size or longer than first (ph2d4/ph1d4 larger than 1.00)

2 - elongated with second phalanx up to 30% shorter than first (ph2d4/ph1d4 between 0.70 - 1.00)

3 - elongated with second phalanx more than 30% shorter than first (ph2d4/ph1d4 smaller than 0.70)

83. Proportional length of the third phalanx of manual digit IV relative to the first phalanx of manual digit IV (ph3d4/ph1d4):

0 - both short or absent

1 - ph3d4 about the same length or larger than ph1d4

2 - ph3d4 shorter than ph1d4

84. Proportional length of the third phalanx of manual digit IV relative to the second phalanx of manual digit IV (ph3d4/ph2d4):

0 - both short or absent

1 - ph3d4 about the same size or longer than ph2d4

2 - ph3d4 shorter than ph2d4

85. Length of metatarsal III:

0 - more than 30% of tibia length

1 - less than 30% of tibia length

86. Fifth pedal digit:

0 - with four phalanges

1 - with 2 phalanges

2 - with 1 or no phalanx (extremely reduced)

87. Last phalanx of pedal digit V:

0 - reduced or absent

1 - elongated, straight

2 - elongated, curved

3 - elongated, very curved (boomerang shape)

88. First phalanx of pedal digit V, length relative to that of metatarsal III (new character):

0 - no less than a third length of metatarsal III (pph1d5/mtIII ≥ 1/3)

1 - less than a third length of metatarsal III (pph1d5/mtIII < 1/3)


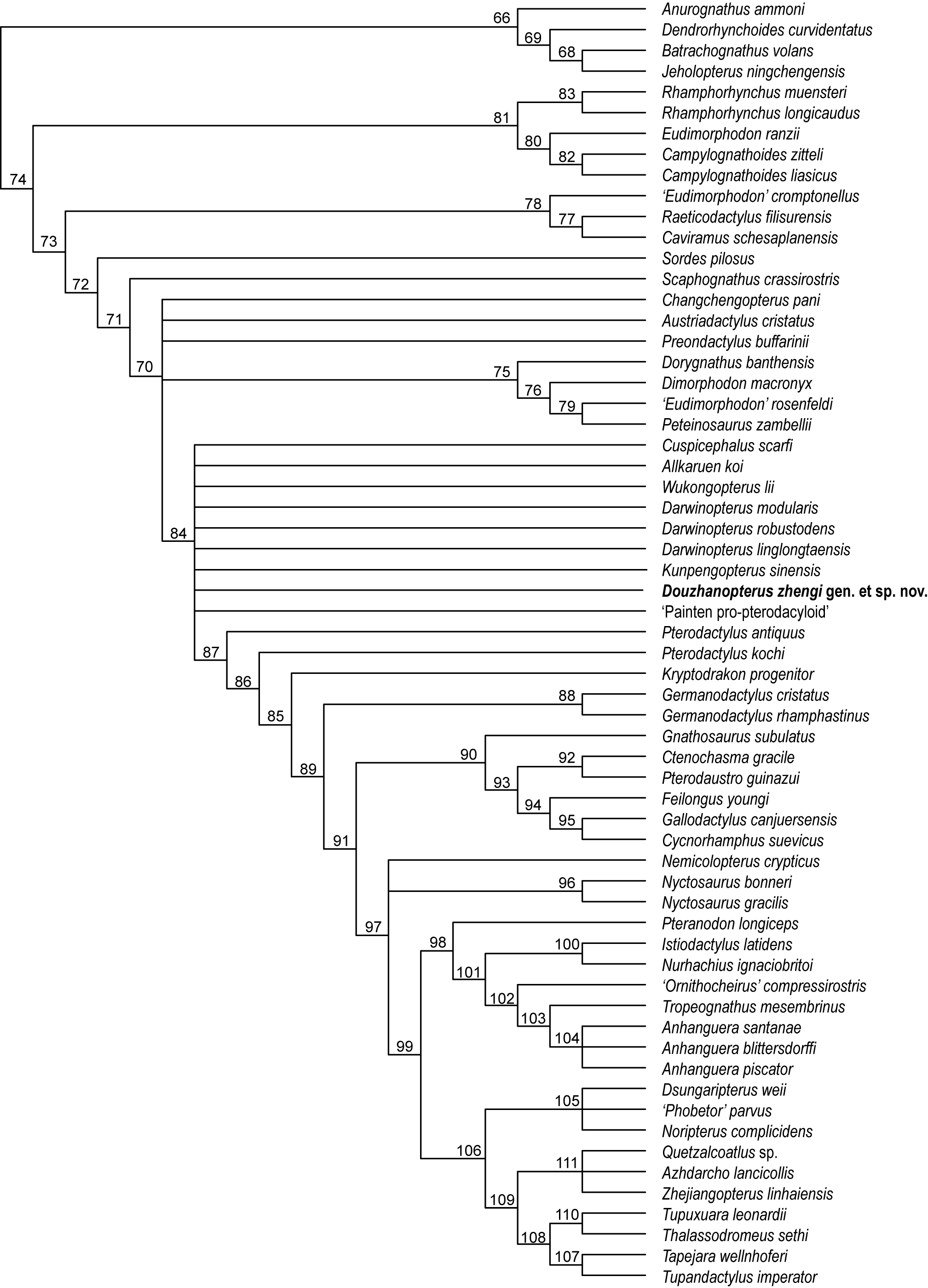


**Supplementary Figure 1. The strict consensus tree of 114 most parsimonious trees with 253.393 steps each.** Synapomorphies are listed below for the nodes present in the strict consensus tree.

Node 66 :

Char. 0: 0.098-0.181 --> 0.004

Char. 3: 0 --> 1

Char. 7: 0 --> 1

Char. 50: 0 --> 1

Char. 60: 0 --> 1

Node 68 :

Char. 1: 0.061 --> 0.068

Node 69 :

Char. 0: 0.004 --> 0.002

Char. 69: 1 --> 2

Node 70 :

Char. 0: 0.456 --> 0.488-0.507

Char. 9: 0 --> 1

Node 71 :

Char. 0: 0.405 --> 0.456

Node 72 :

Char. 82: 2 --> 1

Node 73 :

Char. 80: 1 --> 2

Char. 83: 2 --> 1

Char. 84: 2 --> 1

Node 74 :

Char. 0: 0.098-0.181 --> 0.405

Char. 1: 0.053-0.061 --> 0.115

Char. 4: 0 --> 1

Char. 6: 0 --> 1

Char. 61: 0 --> 1

Node 75 :

Char. 0: 0.488-0.507 --> 0.631

Node 76 :

Char. 0: 0.631 --> 0.787

Char. 1: 0.115-0.128 --> 0.113

Char. 76: 0 --> 1

Char. 87: 3 --> 1

Node 77 :

Char. 43: 0 --> 1

Node 78 :

Char. 48: 0 --> 1

Node 79 :

Char. 48: 0 --> 1

Node 80 :

Char. 40: 0 --> 1

Node 81 :

Char. 36: 0 --> 1

Char. 76: 0 --> 1

Char. 81: 1 --> 2

Node 82 :

Char. 9: 0 --> 1

Char. 11: 0 --> 1

Char. 23: 0 --> 1

Node 83 :

Char. 9: 0 --> 2

Char. 29: 0 --> 1

Char. 39: 0 --> 1

Char. 41: 0 --> 1

Char. 87: 0 --> 2

Node 84 (Monofenestrata):

Char. 0: 0.488-0.507 --> 0.565

Char. 1: 0.115-0.128 --> 0.164

Char. 10: 0 --> 1

Char. 20: 0 --> 2

Char. 21: 0 --> 1

Char. 27: 0 --> 1

Char. 28: 01 --> 2

Char. 29: 0 --> 1

Char. 50: 0 --> 1

Char. 57: 0 --> 1

Char. 74: 0 --> 1

Char. 87: 3 --> 2

Node 85 :

Char. 1: 0.298-0.302 --> 0.312

Node 86 :

Char. 78: 1 --> 2

Node 87 (Pterodactyloidea):

Char. 0: 0.565 --> 0.047-0.063

Char. 1: 0.164 --> 0.298-0.302

Char. 22: 1 --> 0

Char. 39: 0 --> 1

Char. 50: 1 --> 2

Char. 58: 0 --> 1

Char. 60: 0 --> 1

Char. 61: 1 --> 0

Char. 73: 1 --> 7

Char. 74: 1 --> 0

Char. 76: 0 --> 1

Char. 80: 2 --> 3

Char. 82: 1 --> 2

Char. 83: 1 --> 2

Char. 84: 1 --> 2

Char. 86: 1 --> 2

Char. 87: 2 --> 0

Char. 88: 0 --> 1

Node 88 :

Char. 16: 0 --> 4

Node 89 :

Char. 1: 0.312 --> 0.332-0.335

Node 90 :

Char. 4: 1 --> 2

Node 91 :

Char. 50: 2 --> 0

Node 92 :

Char. 1: 0.332-0.523 --> 0.321

Char. 51: 0 --> 1

Char. 85: 1 --> 0

Node 93 :

Char. 2: 0 --> 1

Node 94 :

Char. 26: 0 --> 2

Char. 44: 0 --> 2

Node 95 :

Char. 4: 2 --> 1

Char. 21: 1 --> 2

Node 96 :

Char. 73: 7 --> 5

Char. 81: 1 --> 2

Node 97 :

Char. 1: 0.332-0.523 --> 0.607

Char. 21: 1 --> 0

Char. 27: 1 --> 0

Char. 30: 0 --> 1

Char. 36: 0 --> 1

Char. 44: 0 --> 3

Char. 54: 0 --> 1

Char. 55: 0 --> 1

Char. 57: 1 --> 0

Char. 59: 2 --> 0

Node 98 :

Char. 59: 0 --> 1

Char. 62: 0 --> 1

Char. 63: 0 --> 1

Char. 73: 7 --> 6

Char. 75: 0 --> 1

Node 99 :

Char. 26: 0 --> 3

Char. 56: 0 --> 1

Node 100 :

Char. 10: 1 --> 2

Char. 24: 0 --> 2

Char. 52: 0 --> 1

Node 101 :

Char. 1: 0.607 --> 0.339

Char. 64: 0 --> 1

Char. 76: 1 --> 2

Node 102 :

Char. 35: 0 --> 1

Node 103 :

Char. 16: 0 --> 1

Char. 18: 0 --> 1

Char. 42: 0 --> 1

Node 104 :

Char. 46: 0 --> 1

Node 105 :

Char. 12: 0 --> 1

Char. 15: 0 --> 1

Char. 16: 0 --> 3

Char. 19: 0 --> 1

Char. 44: 3 --> 1

Char. 45: 0 --> 1

Char. 47: 0 --> 1

Node 106 :

Char. 31: 0 --> 1

Char. 33: 0 --> 1

Char. 69: 1 --> 0

Char. 70: 1 --> 0

Node 107 :

Char. 35: 0 --> 3

Node 108 :

Char. 1: 0.607 --> 0.508

Char. 10: 1 --> 2

Char. 13: 0 --> 1

Char. 16: 0 --> 5

Char. 24: 0 --> 1

Char. 67: 0 --> 1

Node 109 :

Char. 14: 0 --> 1

Char. 82: 2 --> 3

Node 110 :

Char. 5: 0 --> 1

Node 111 :

Char. 1: 0.607 --> 0.678

Char. 56: 1 --> 0

Char. 57: 0 --> 2

Char. 59: 0 --> 3

Char. 66: 0 --> 1

**Supplementary Table 1. The measurements plotted in Figure 3A (in mm).** Abbreviation: hu, length of humerus; ta, length of tail.

| Taxa | hu | ta | Sources |
| --- | --- | --- | --- |
| *Douzhanopterus zhengi* gen. et sp. nov. holotype | 48.49 | 83.9 | Measured by authors |
| ‘Painten pro-pterodactyloid’ | 34.3 | 24.4* | 14 |
| *Dendrorhynchoides mutoudengensis* holotype | 27 | 18* | S1 |
| *Dendrorhynchoides curvidentatus* holotypr | 27.8 | 9.2* | S2, S3 |
| *Anurognathus ammoni* (private collection) | 18.2 | 7.3 | S4 |
| *Batrachognathus volans* PIN 2585/4A | 30 | 44* | S5 |
| Anurognthid IVPP V 16728 | 28.2 | 29.3* | S3 |
| *Wukongopterus lii* holotype | 38.7 | 151.8* | Measured by authors |
| *Darwinopterus modularis* holotype | 59 | 287* | 7 |
| *Darwinopterus modularis* YH-2000 | 44 | 205 | 7 |
| *Darwinopterus robustodens* holotype | 50 | 230* | 9 |
| *Jianchangopterus zhaoianus* holotype | 21.4 | 68.3* | S6 |
| *Pterorhynchus wellnhoferi* holotype | 5.2 | 40.2* | S7 |
| *Dorygnathus* BSP 1938 I 49 | 50 | 198* | Measured on photos by authors |
| *Rhamphorhynchus* sp. no number | 39 | 493* | Measured on photos by authors |
| *Rhamphorhynchus gemmini* BSP no number | 60 | 269* | Measured on photos by authors |
| *Rhamphorhynchus longicaudus* BSP 1877 X 1 | 14 | 94.8 | Measured on photos by authors |
| *Rhamphorhynchus longicaudus* BSP 1889 XI 1 | 16.3 | 107* | Measured on photos by authors |
| *Rhamphorhynchus longicaudus* BSP 1938 I 503 | 14 | 132 | Measured on photos by authors |
| *Scaphognathus crassirostris* SMNS 59395 | 25.8 | 112.5 | Measured on photos by authors |
| *Rhamphorhynchus* SOS 2819 | 38.7 | 253 | Measured on photos by authors |
| *Sordes pilosus* holotype | 40 | 254.1 | S1, S8 |
| *Boreopterus cuiae* holotype | 79 | 75 | S1, S9 |
| *Gegepterus changae* IVPP V 11972 | 22.4† | 9.8 | S10 |
| *Zhenyuanopterus longirostris* holotype | 210 | 130 | S11 |
| *Pterodactylus antiquus* holotype | 31.5 | 21 | 25 |
| *Pterodactylus kochi* PTH No. 42736 | 14 | 8.8† | 25 |
| *Pterodactylus kochi* holotype | 28.5 | 15 | S12 |
| *Pterodactylus micronyx* neotype | 25 | 17 | 25 |
| *Pterodactylus elegans* neotype | 15 | 5 | 25 |
| *Pterodaustro guinazui* MHIN-UNSL-GEO-V 243 | 19.23 | 14.56* | S13 |
| *Anhanguera pisctors* holotype | 255 | 165* | S14 |
| *Pteranodon* composite specimen | 253 | 190 | S15 |
| *Zhejiangopterus linhaiensis* ZNHM M1323 | 130 | 24 | S16 |

*Tails without some distal most caudal vertebrae.

†Estimated length.

**Supplementary Table 2. The measurements plotted in Figure 3B (in mm).** Abbreviation: hu, length of humerus; mcIV, length of wing metacarpal; ul, length of ulna.

| Taxa | hu | ul | mcIV | mcIV/ul | Sources |
| --- | --- | --- | --- | --- | --- |
| *Douzhanopterus zhengi* gen. et sp. nov. holotype | 48.49 | 59.49 | 31.72 | 0.53 | Measured by authors |
| ‘Painten pro-pterodactyloid’ | 34.3 | 46.5 | 21.6 | 0.46 | 14 |
| *Dendrorhynchoides mutoudengensis* holotype | 27 | 42 | 11 | 0.26 | S1 |
| *Jeholopterus ningchengensis* holotype | 62 | 89 | 19* | 0.21 | S17 |
| *Dendrorhynchoides curvidentatus* holotype | 27.8 | 35.5 | 9.3 | 0.26 | S2 |
| *Wukongopterus lii* holotype | 38.7 | 62.1 | 22.9 | 0.37 | 6 |
| *Kunpengopterus sinensis* holotype | 36.2* | 59.2* | 23* | 0.39 | 8 |
| *Darwinopterus modularis* holotype | 59 | 87 | 36 | 0.41 | 7 |
| *Darwinopterus modularis* YH-2000 | 44 | 64 | 26 | 0.41 | 7 |
| *Darwinopterus linglongtaensis* holotype | 40.4 | 58 | 22.5 | 0.39 | 8 |
| *Darwinopterus robustodens* holotype | 50 | 80 | 30 | 0.38 | 9 |
| *Changchengopterus pani* holotype | 25.9 | 36.8 | 14.5 | 0.39 | S18 |
| *Changchengopterus pani* PMOL-AP00010 | 50.2 | 76 | 27 | 0.36 | S19 |
| *Qinglongopterus guoi* holotype | 17.8 | 28.3 | 9.1 | 0.32 | S20 |
| *Fenghuangopterus lii* holotype | 63 | 85 | 35 | 0.41 | S21 |
| *Eudimorphodon ranzii* holotype | 47 | 65 | 29 | 0.45 | S8 |
| *Dorygnathus banthensis* PMR 156 | 61 | 96 | 29 | 0.30 | S8 |
| *Rhamphorhynchus longicaudus* holotype | 16.5 | 26.7 | 10 | 0.37 | S8 |
| *Rhamphorhynchus intermius* holotype | 19.5 | 30 | 11.8 | 0.39 | S8 |
| *Rhamphorhynchus muensteri* SMF R4128 | 38 | 66 | 21 | 0.32 | S8 |
| *Rhamphorhynchus gemmingi* UHMYE 13 | 38.5 | 61 | 18 | 0.30 | S8 |
| *Campylognathoides liasicus* CM 11424 | 50.3 | 59.5 | 23 | 0.39 | S8 |
| *Campylognathoides zitteli* holotype | 70 | 82 | 30 | 0.37 | S8 |
| *Scaphognathus crassirostris* holotype | 53.5 | 94 | 27 | 0.29 | S8 |
| *Sordes pilosus* holotype | 40 | 68 | 15 | 0.22 | S8 |
| *Nesodactylus hesperius* holotype | 46.5 | 79 | 26.7 | 0.34 | S8 |
| *Anurognathus ammoni* holotype | 32 | 45 | 11 | 0.24 | S8 |
| *Sinopterus dongi* IVPP V 13363 | 58 | 87.5 | 95 | 1.09 | 26 |
| *Sinopterus dongi* DNHM D2525 | 108 | 154 | 169 | 1.10 | S22 |
| *Chaoyangopterus zhangi* holotype | 93 | 133 | 185 | 1.39 | S23 |
| *Istiodactylus sinensis* holotype | 133.5 | 233.7 | 162.3 | 0.69 | S24 |
| *Longchengpterus zhaoi* holotype | 88 | 147 | 100 | 0.68 | S25 |
| *Forfexopterus jeholensis* holotype | 117.6 | 192.2 | 146.4 | 0.76 | S26 |
| *Shenzhoupterus chaoyangensis* holotype | 66 | 105 | 140 | 1.33 | S27 |
| *Eosipterus yangi* DNHM D2514 | 47 | 60 | 42 | 0.70 | S28 |
| *Haopterus gracilis* holotype | 70 | 101 | 89 | 0.88 | S29 |
| *Beipiaopterus chenianus* holotype | 68 | 75 | 75 | 1.00 | S30 |
| *Boreopterus cuiae* holotype | 79 | 110 | 94 | 0.85 | S9 |
| *Elanodactylus prolatus* holotype | 151.3 | 154.3* | 126.5* | 0.82 | S31 |
| *Ningchengopterus liuae* holotype | 15 | 20 | 15 | 0.75 | S32 |
| *Zhenyuanopterus longirostris* holotype | 210 | 262 | 230 | 0.88 | S11 |
| *Huaxiapterus jii* holotype | 79 | 117 | 132 | 1.13 | S33 |
| *Huaxiapterus corollatus* holotype | 79.7 | 114 | 152 | 1.33 | S34 |
| *Huaxiapterus benxiensis* holotype | 62 | 119 | 133 | 1.12 | S35 |
| *Jidapterus endentus* holotype | 80 | 110 | 140 | 1.27 | S36 |
| *Eoazhdarcho liaoxiensis* holotype | 90 | 122 | 135 | 1.11 | S37 |
| *Pterodactylus kochi* holotype | 28.5 | 41.5 | 30.5 | 0.73 | S8 |
| *Pterodactylus antiquus* holotype | 31.5 | 47 | 35 | 0.74 | S8 |
| *Pterodactylus micronyx* holotype | 25 | 31 | 40 | 1.29 | S8 |
| *Pterodactylus elegans* holotype | 15 | 20 | 16.5 | 0.83 | S8 |
| *Pterodactylus longicollum* holotype | 78 | 104 | 130 | 1.25 | S8 |
| *Gernanodactylus cristatus* holotype | 56 | 75 | 66 | 0.88 | S8 |
| *Gernanodactylus rhamphastinus* holotype | 60 | 100* | 70 | 0.70 | S8 |
| *Ctenochasma gracile* holotype | 38.5 | 52.5 | 35 | 0.67 | S8 |
| *Pterodaustro guinazui* PVL 3860 | 80 | 102 | 78 | 0.76 | S8 |
| *Gallodactylus canjuersensis* holotype | 48 | 56 | 86* | 1.54 | S8 |
| *Cycnorhamphus suevicus* holotype | 65.5 | 87 | 108 | 1.24 | S8 |
| *Noripterus complicidens* 64043-3 | 77 | 98 | 140 | 1.43 | S8 |
| *Anhanguera pisctors* holotype | 255 | 390 | 256 | 0.66 | S14 |

*Estimated length.

**Supplementary Table 3. The measurements plotted in Figure 3C (in mm).** Abbreviation: mtIII, length of metatarsal III; pph1d5, length of first phalange of pedal digit 5.

| Taxa | pph1d5 | mtIII | Sources |
| --- | --- | --- | --- |
| *Douzhanopterus zhengi* gen. et sp. nov. holotype | 4.54 | 21.58 | Measured by authors |
| ‘Painten pro-pterodactyloid’ | 3.8 | 12.1 | 4 |
| *Pterodactlyus elegens* neotype | 0.6 | 7 | 25 |
| *Beipiaopterus chenianus* holotype | 3.5 | 38 | S30 |
| *Gegepterus changae* IVPP V 11972 | 2.4 | 25.8 | S10 |
| *Pterodactylus* sp. no number | 2.5* | 22.4* | S38, Fig. 3c |
| *Pterodactylus mixronyx* holotype | 1.3 | 9.5 | 25 |
| *Pterodactylus antiquus* holotype | 2.2 | 16 | 25 |
| *Campylognathus liasicus* CM 11424 | 6 | 21.6 | S39 |
| *Rhamphorhynchus longicaudus* holotype | 3.6* | 9.5*42 | S40, Fig. 17a |
| *Qinglongopterus guoi* holotype | 3 | 7.1 | S21 |
| *Kunpengopterus sinensis* holotype | 11.2 | 22 | Measured by authors |
| *Carniadactylus rosenfeldi* holotype | 13.5 | 21 | S41 |
| *Rhamphorhynchus munsteri* TM no. 6921 | 15.4* | 23.6* | S40, Fig. 17e |
| *Anurognathus ammoni* (private collection) | 6 | 8.5 | S4 |
| *Darwinopterus linglongtaensis* holotype | 12.5 | 17 | Measured by authors |
| *Orientognathus chaoyngensis* holotype | 25.6 | 35 | S42 |
| *Wukongopterus lii* holotype | 13.7 | 17 | Measured by authors |
| *Preondactylus buffarinii* holotype | 12.6* | 15.9* | S43, Fig. 1 |
| *Peteinosaurus zambellii* MCSNB 3359 | 14.7* | 17.9* | S44, Fig. 41 |
| *Dendrorhynchoides mutoudengensis* holotype | 10 | 12 | S11 |
| *Anurognathus ammoni* holotype | 15.3 | 18 | S4 |
| *Changchengopterus pani* holotype | 14.5 | 17 | S19 |
| *Darwinopterus robustodens* holotype | 18 | 21 | 9 |
| *Eudimorphodon ranzii* holotype | 7.1* | 8* | S45, Fig. 12 |
| *Darwinopterus* with eggs ZMNH M8802 | 18.5 | 20 | 10 |
| *Jianchangnathus robustus* holotype | 24.6 | 25.9 | S46 |
| *Sordes pilosus* holotype | 13.6 | 14.3 | S47, Fig. 1d |
| *Scaphognathus crassirostris* SMNS 59395 | 11.5* | 11.1 | S48, Tab. 1 & Fig. 3 |
| *Jeholopterus ningchengensis* holotype | 23 | 22 | S17 |

*Measured on figures in previous study.

Supplementary references:

S1. Lü, J. & Hone, D. W. E. A new Chinese anurognathid pterosaur and the evolution of pterosaurian tail lengths. *Acta Geol. Sin.* **86**, 1317-1325 (2012).

S2. Ji, S. & Ji, Q. A new fossil pterosaur (Rhamphorhynchoidea) from Liaoning. *Jiangsu Geol.* **24**, 199-206 (1998). (in Chinese with English abstrct)

S3. Jiang, S. *et al.* Short note on an anurognathid pterosaur with a long tail from the Upper Jurassic of China. *Hist. Biol.* **27**, 717-721 (2015).

S4. Bennett, S. C. A second specimen of the pterosaur *Anurognathus ammoni*. *Paläont. Zeitschr.* **81**, 376-398 (2007).

S5. Costa, F. R., Alifanov, V., Dalla Vecchia, F. M. & Kellner, A. W. A. in *Rio Ptero 2013 - International Symposium on Pterosaurs.* (eds Sayão, J. M., Costa, F. R., Bantim, R. A. M. & Kellner, A. W. A.) 54-55 (Universidade Federal do Rio de Janeiro, Museu Nacional, 2013).

S6. Lü, J. & Bo, X. A new rhamphorhynchid Pterosaur (Pterosauria) from the Middle Jurassic Tiaojishan Formation of western Liaoning, China. *Acta Geol. Sin.* **85**, 977-983 (2011).

S7. Czerkas, S. A. & Ji, Q. in *Feathered Dinosaurs and the origin of flight.* (ed Czerkas, S. A.) 15-41 (Dinosaur Museum, 2002).

S8. Wellnhofer, P. Pterosauria. *Handbuch der Paläoherpetologie* **19,** x-82 (1978). (in German)

S9. Lü, J. & Ji, Q. A new ornithocheirid from the Early Cretaceous of Liaoning Province, China. *Acta Geol. Sin.* **79**, 157-163 (2005).

S10. Jiang, S. & Wang, X. Important features of *Gegepterus changae* (Pterosauria: Archaeopterodactyloidea, Ctenochasmatidae) from a new specimen. *Vert. PalAsiat.* **49**, 172-184 (2011).

S11. Lü, J. A new Boreopterid pterodactyloid pterosaur from the Early Cretaceous Yixian Formation of Liaoning Province, northeastern China. *Acta Geol. Sin.* **84**, 241-246 (2010).

S12. Wellnhofer, P. Über *Pterodactylus kochi* (Wagner 1837). *N. Jb. Geol. Paläont. Abh.* **132**, 97-126 (1968). (in German with English abstract)

S13. Codorniú, L. S. Morfología caudal de *Pterodaustro guinazui* (Pterosauria: Ctenochasmatidae) del Cretácico de Argentina. *Ameghiniana* **42**, 505-509 (2005). (in Spanish)

S14. Kellner, A. W. A. & Tomida, Y. Description of a new species of Anhangueridae (Pterodactyloidea) with comments on the pterosaur fauna from the Santana Formation (Aptian-Albian), northeastern Brazil. *Nation. Sci. Mus. Mon.* **17**, ix-137 (2000).

S15. Bennett, S. C. The osteology and functional morphology of the Late Cretaceous pterosaur *Pteranodon* Part I. General description of osteology. *Palaeontogr. Abt. A* **260,** 1-112 (2001).

S16. Cai, Z. & Wei, F. on a new pterosaur (*Zhejiangopterus linhaiensis* gen. et sp. nov.) from Upper Cretaceous in Linhai, Zhejiang, China. *Vert. PalAsiat.* **32**, 181-194 (1994). (in Chinese with English summary)

S17. Wang, X., Zhou, Z., Zhang, F. & Xu, X. A nearly completely articulated rhamphorhynchoid pterosaur with exceptionally well-preserved wing membranes and “hairs” from Inner Mongolia, northeast China. *Chinese Sci. Bull.* **47**, 226-230 (2002).

S18. Lü, J. A new non-pterodactyloid pterosaur from Qinglong County, Hebei Province of China. *Acta Geol. Sin.* **83**, 189-199 (2009).

S19. Zhou, C. & Schoch, R. R. New material of the non-pterodactyloid pterosaur *Changchengopterus pani* Lü, 2009 from the Late Jurassic Tiaojishan Formation of western Liaoning. *N. Jb. Geol. Paläont. Abh.* **260**, 265-275 (2011).

S20. Lü, J., Unwin, D. M., Zhao, B., Gao, C. & Shen, C. A new rhamphorhynchid (Pterosauria: Rhamphorhynchidae) from the Middle/Upper Jurassic of Qinglong, Hebei Province, China. *Zootaxa* **3158**, 1-19 (2012).

S21. Lü, J., Fucha, X. & Chen, J. A new scaphognathine pterosaur from the Middle Jurassic of western Liaoning, China. *Acta Geosci. Sin.* **31**, 263-266 (2010).

S22. Lü, J. *et al.* New material of pterosaur *Sinopterus* (Reptilia : Pterosauria) from the Early Cretaceous Jiufotang Formation, Western Liaoning, China. *Acta Geol. Sin.* **80**, 783-789 (2006).

S23. Wang, X. & Zhou, Z. Two new pterodactyloid pterosaurs from the Early Cretaceous Jiufotang Formation of western Liaoning, China. *Vert. PalAsiat.* **41**, 34-41 (2003).

S24. Andres, B. & Ji, Q. A new species of *Istiodactylus* (Pterosauria, Pterodactyloidea) from the Lower Cretaceous of Liaoning, China. *J. Vert. Paleont.* **26**, 70-78 (2006).

S25. Wang, L., Li, L., Duan, Y. & Cheng, S. A new istiodactylid pterosaur from western Liaoning. *Geol. Bull. China* **25**, 737-740 (2006).

S26. Jiang, S., Cheng, X., Ma, Y. & Wang, X. A new archaeopterodactyloid pterosaur from the Jiufotang Formation of western Liaoning, China, with a comparison of sterna in Pterodactylomorpha. *J. Vert. Paleont.* doi:10.1080/02724634.2016.1212058 (2016).

S27. Lü, J., Unwin, D. M., Xu, L. & Zhang, X. A new azhdarchoid pterosaur from the Lower Cretaceous of China and its implications for pterosaur phylogeny and evolution. *Naturwissenschaften* **95**, 891-897 (2008).

S28. Lü, J., Gao, C., Meng, Q., Liu, j. & Ji, Q. On the systematic position of *Eosipterus yangi* Ji et Ji, 1997 among pterodactyloids. *Acta Geol. Sin.* **80**, 643-646 (2006).

S29. Wang, X. & Lü, J. Discovery of a pterodactylid pterosaur from the Yixian Formation of western Liaoning, China. *Chinese Sci. Bull.* **46**, 1112-1117 (2001).

S30. Lü, J. A new pterosaur: *Beipiaopterus chenianus*, gen. et sp. nov. (reptilia: pterosauria) from western Liaoning Province of China. *Mem. Fukui Pref. Dinosaur Mus.* **2**, 153-160 (2003).

S31. Andres, B. & Ji, Q. A new pterosaur from the Liaoning Province of China, the phylogeny of the Pterodactyloidea, and convergence in their cervical vertebrae. *Palaeontology* **51**, 453-469 (2008).

S32. Lü, J. A baby pterodactyloid pterosaur from the Yixian Formation of Ningcheng, Inner Mongolia, China. *Acta Geol. Sin.* **83**, 1-8 (2009).

S33. Lü, J. & Yuan, C. New tapejarid pterosaur from Western Liaoning, China. *Acta Geol. Sin.* **79**, 453-458 (2005).

S34. Lü, J. *et al.* A new species of *Huaxiapterus* (Pterosauria : Pterodactyloidea) from the Lower Cretaceous of western Liaoning, China with comments on the systematics of tapejarid pterosaurs. *Acta Geol. Sin.* **80**, 315-326 (2006).

S35. Lü, J., Gao, Y., Xing, L., Li, Z. & Ji, Q. A new species of *Huaxiapterus* (Pterosauria : Tapejaridae) from the Early Cretaceous of western Liaoning, China. *Acta Geol. Sin.* **81**, 683-687 (2007).

S36. Dong, Z., Sun, Y. & Wu, S. On a new pterosaur from the Lower Cretaceous of Chaoyang Basin, western Liaoning, China. *Glob. Geol.* **22**, 1-7 (2003). (in Chinese with English abstract)

S37. Lü, J. & Ji, Q. New azhdarchid pterosaur from the Early Cretaceous of western Liaoning. *Acta Geol. Sin.* **79**, 301-307 (2005).

S38. Frey, E. & Tischlinger, H. Weichteilanatomie der Flugsaurierfüße und Bau der Scheitelkämme: Neue Pterosaurierfunde aus den Solnhofener Schichten (Bayern) und der Crato-Formation (Brasilien). *Archaeopteryx* **18**, 1-16 (2000). (in German with English abstract)

S39. Wellnhofer, P. *Campylognathoides liasicus* (Quenstedt), an Upper Liassic pterosaur from Holzmaden: the Pittsburgh specimen. *Ann. Carnegie Mus.* **147**, 5-34 (1974).

S40. Wellnhofer, P. Die Rhamphorhynchoidea (Pterosauria) der Oberjura-plattenkalke süddeutschands Teil I. *Palaeontogr. Abt. A* **148**, 1-33 (1975). (in German)

S41. Dalla Vecchia, F. M. Anatomy and systematics of the pterosaur *Carniadactylus* gen. n. *rosenfeldi* (Dalla Vecchia, 1995). *Riv. It. Paleont.Strat.* **115** (2009).

S42. Lü, J. *et al.* A new rhamphorhynchid pterosaur (Pterosauria) from Jurassic deposits of Liaoning Province, China. *Zootaxa* **3911**, 119-129 (2015).

S43. Dalla Vecchia, F. M. New observations on the osteology and taxonomic status of *Preondactylus buffarinii* Wild, 1984 (Reptilia, Pterosauria). *Boll. Soc. Paleont. It.* **36**, 1997 (1998).

S44. Wild, R. Die Flugsaurier (Reptilia, Pterosauria) aus der Oberen Trias von Cene bei Bergamo, Italien. *Boll. Soc. Paleont. It.* **17**, 176-256 (1978). (in German with English summary)

S45. Wild, R. A juvenile specimen of *Eudimorphodon ranzii* Zambelli (Reptilia, Pterosauria) from the upper Triassic (Norian) of Bergamo. *Riv. Mus. Civ. Sci. Nat.* **16**, 95-120 (1993).

S46. Cheng, X., Wang, X., Jiang, S. & Kellner, A. W. A. A new scaphognathid pterosaur from western Liaoning, China. *Hist. Biol.* **24**, 101-111 (2012).

S47. Unwin, D. M. & Bakhurina, N. N. *Sordes pilosus* and the nature of the pterosaur flight apparatus. *Nature* **371**, 62-62 (1994).

S48. Bennett, S. C. A new specimen of the pterosaur *Scaphognathus crassirostris*, with comments on constraint of cervical vertebrae number in pterosaurs. *N. Jb. Geol. Paläont. Abh.* **271**, 327-348 (2014).
